# GST‐IVTT pull‐down: a fast and versatile *in vitro* method for validating and mapping protein–protein interactions

**DOI:** 10.1002/2211-5463.13485

**Published:** 2022-09-22

**Authors:** Zsuzsánna Réthi‐Nagy, Edit Ábrahám, Zoltán Lipinszki

**Affiliations:** ^1^ Biological Research Centre, Institute of Biochemistry, MTA SZBK Lendület Laboratory of Cell Cycle Regulation ELKH Szeged Hungary; ^2^ Doctoral School of Biology, Faculty of Science and Informatics University of Szeged Hungary

**Keywords:** GST pull‐down, IVTT, mapping, protein–protein interaction, SLiMs

## Abstract

Over the past few decades, dozens of *in vitro* methods have been developed to map, investigate and validate protein–protein interactions. However, most of these approaches are time‐consuming and labour‐intensive or require specialised equipment or substantial amounts of purified proteins. Here, we describe a fast and versatile research protocol that is suitable for the *in vitro* analysis of the physical interaction between proteins or for mapping the binding surfaces. The principle of this method is based on the immobilisation of the protein/domain of interest to a carrier followed by its incubation with a labelled putative binding partner, which is generated by a coupled *in vitro* transcription/translation reaction. Interacting proteins are removed from the carrier, fractionated and visualised by SDS/PAGE autoradiography (or western blotting). This simple and cheap method can be easily carried out in every wet lab.

## Graphical abstract

The GST‐IVTT pull‐down assay is suitable for the rapid validation and mapping of functional protein interactions *in vitro*. The ^35^S‐labelled prey protein or its pieces (putative interactors) are produced by a single‐step IVTT (*in vitro* transcription/translation) reaction from various templates. Preys are mixed with immobilised GST (control) or GST‐tagged proteins of interest, and the binding is simply analysed by SDS/PAGE autoradiography.

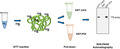

**Abbreviations**
3′: three‐prime end of DNA
^35^S: the radioactive isotope of sulphur with relative atomic mass 35
5′: five‐prime end of DNA
aa: amino acid
Ana2: Anastral spindle 2
ATG: start codon
AxxA: alanine‐any amino acid‐any amino acid‐alanine motif
BB: binding buffer
BSA: bovine serum albumin
cDNA: complementary DNA
CDS: coding sequence
CENP‐C: Centromeric protein‐C
ctrl: control
DNA: deoxyribonucleic acid
DTT: dithiothreitol
*E. coli*
: *Escherichia coli*
EDTA: ethylenediaminetetraacetic acid
EGTA: ethylene glycol‐bis(β‐aminoethyl ether)‐*N*,*N*,*N*′,*N*′‐tetraacetic acid
EVH1: enabled/vasodilator‐stimulated phosphoprotein homology 1 domain
FBD: Falafel‐binding domain
Flfl^N^
: the N‐terminal half of Falafel
FxxP: phenylalanine‐any amino acid‐any amino acid‐proline motif
GST: Glutathione S‐transferase
h: hour(s)
HEPES: 4‐(2‐hydroxyethyl)‐1‐piperazineethanesulfonic acid
HF: high fidelity
IMAC: immobilised‐metal affinity chromatography
IPTG: Isopropyl β‐D‐1‐thiogalactopyranoside
IVTT: *in vitro* transcription and translation reaction
KD: kinase dead
kDa: kilodalton
LB: Luria‐Bertani medium
max: maximum
min: minute
mRNA: messenger ribonucleic acid
MxPP: methionine‐any amino acid‐proline‐proline motif
PBS: phosphate‐buffered saline
PCR: polymerase chain reaction
PIC: protease inhibitor cocktail
Plk4: Polo‐like kinase 4
PMSF: phenylmethylsulfonyl fluoride
POI: protein of interest
PP4: Protein phosphatase 4
PVDF: polyvinylidene fluoride
R3: the R3 scaffold subunit of PP4
RT: room temperature
Sas6: Spindle assembly abnormal 6
SDS: sodium dodecyl sulphate
SDS/PAGE: sodium dodecyl sulphate polyacrylamide gel electrophoresis
SLiM: short linear motif
TAE: Tris Acetate‐EDTA buffer
Tris: Tris(hydroxymethyl)aminomethane
Triton X‐100: nonionic surfactant, polyethylene glycol mono(4‐tert‐octylphenyl) ether
UTR: untranslated region
WB1‐2: wash buffer 1–2

Investigating protein–protein interactions is key to understanding the function of protein complexes in cells. Over the years many approaches have been developed to validate the physical interaction between proteins. This includes the classical pull‐down assay and co‐immunoprecipitation, or more sophisticated techniques such as yeast two/three hybrid screen, bimolecular fluorescence complementation, microscale thermophoresis, fluorescence resonance energy transfer, etc. [1]. Undoubtedly, these are very useful methods, however, time‐consuming and resource‐intensive. When the direct binding of a large number of potential interacting partners of the protein of interest (POI) needs to be tested, a simpler and faster method is more beneficial. Among these, we use a noncanonical GST pull‐down assay, in which the protein/domain/fragment of interest (hereafter bait) is immobilised onto a carrier and mixed with a labelled putative interactor protein or its derivatives (hereafter prey).

We usually generate the recombinant POI fused to Glutathione S‐transferase domain [2] (hereafter GST‐POI) in bacteria followed by its purification and immobilisation onto Glutathione Sepharose beads. However, other affinity tags and matrices (e.g. Polyhistidine/IMAC [3, 4]) can also be used. Once a good quality of immobilised GST‐POI is available, dozens of prey candidates can be generated and tested for direct binding in a short period of time.

Prey proteins are expressed in a single‐step, coupled *in vitro* transcription and eukaryotic translation reaction (IVTT) from various template DNAs. We use the T7 promoter‐driven reticulocyte‐derived hybrid IVTT system; however, other IVTT kits are also available. Templates can be plasmids, but also linear DNA fragments made by PCR. For efficient detection, we label the prey proteins with radioactive ^35^S‐methionine; however, other labelling strategies may also apply. Finally, the prey is mixed with the immobilised GST‐POI and bound proteins are analysed by SDS/PAGE autoradiography (or western blotting; Fig. 1).

**Fig. 1:**
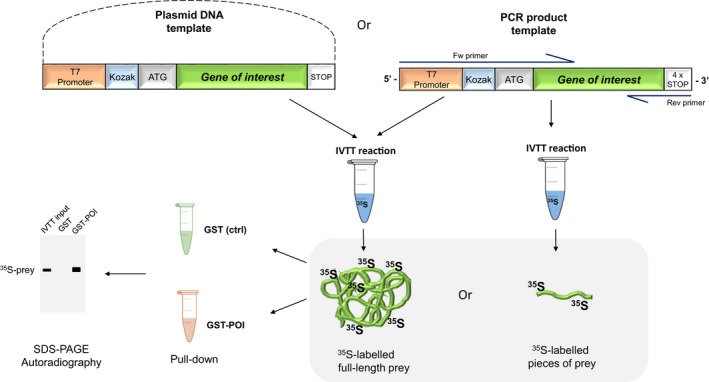
Schematic representation of the GST‐IVTT pull‐down assay. IVTT is performed on plasmids or PCR‐produced DNA encoding the putative full‐length, truncated or mutated prey protein. The ^35^S‐labelled prey is incubated with GST (ctrl) or GST‐POI baits, respectively, followed by the visualisation of the binding by SDS/PAGE autoradiography.

With this approach, we successfully validated the direct binding between several baits and affinity‐purification coupled to mass spectrometry‐identified putative interactors [3, 4, 5, 6, 7, 8], or narrowed‐down the interacting domains/regions of proteins (Fig. 2) [5, 8, 9]. In addition, we used this protocol to prove the phosphorylation‐dependent interaction between various polypeptides (Fig. 3) [10, 11] and identified short linear motifs (SLiMs) or single amino acids that are involved in the direct binding between an enzyme and its substrates (Fig. 4) [5, 8].

**Fig. 2:**
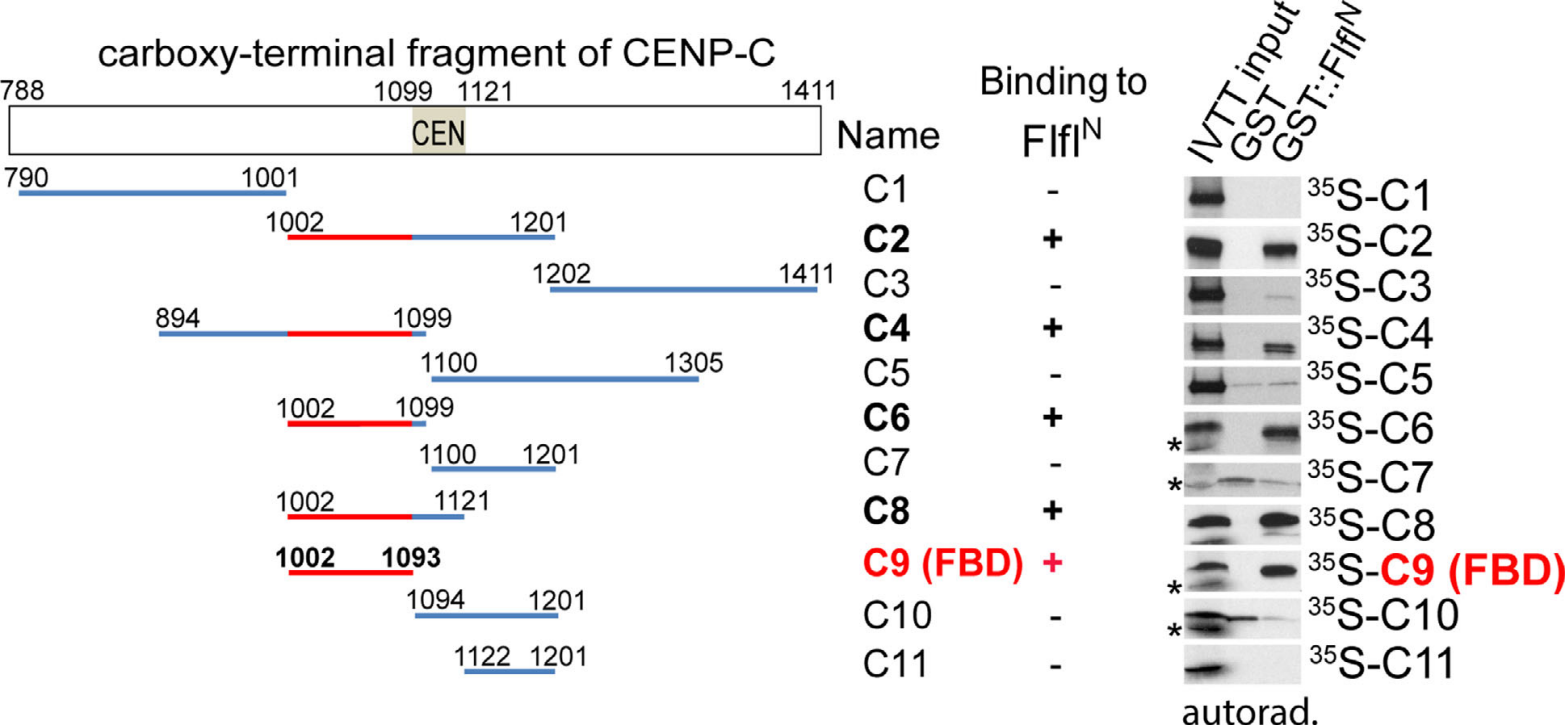
Mapping the Falafel‐binding domain (FBD) of CENP‐C. Immobilised GST (negative control) or GST‐tagged N‐terminal region (aa 1–361) of Falafel (GST‐Flfl^N^), the R3 subunit of the *Drosophila* PP4 phosphatase, was incubated with ^35^S‐methionine‐labelled overlapping pieces of the C‐terminal half of the key centromeric protein, CENP‐C [12] (C1‐C11 fragments; templates were generated by PCR). Interactions were analysed by SDS/PAGE autoradiography, which revealed that the 92 aa‐long C9 fragment (indicated in red) of CENP‐C is the FBD. The figure was adapted from [5].

**Fig. 3:**
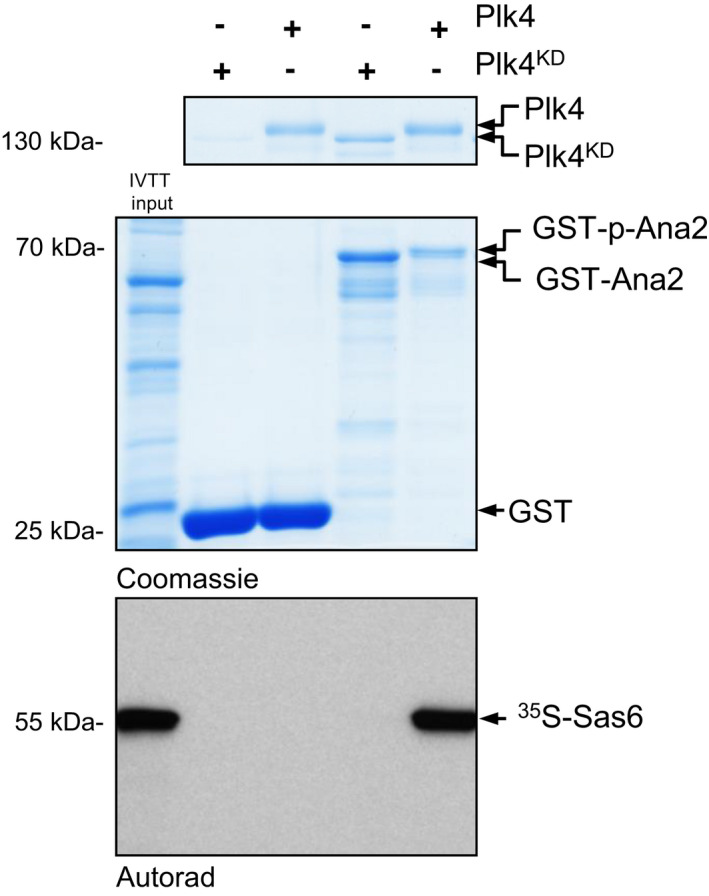
Plk4 kinase phosphorylates Ana2, a prerequisite for Sas6 binding to Ana2. Immobilised GST (negative control) or GST‐tagged Ana2 were treated with either active Plk4 or kinase dead Plk4^KD^ and incubated *in vitro* with ^35^S‐methionine‐labelled Sas6. The resulting complex was analysed by SDS/PAGE (Coomassie) and autoradiography, which revealed that Sas6 specifically interacts with Ana2, but only when Ana2 is prephosphorylated by Plk4. Figure is adapted from [11].

**Fig. 4:**
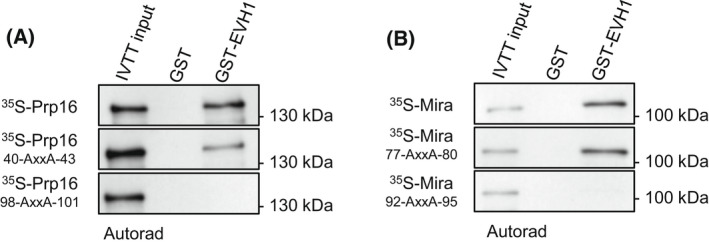
Identification of the Falafel's EVH1 domain‐binding FxxP or MxPP short linear motifs (SLiMs). Autoradiography images of *in vitro* binding of GST‐Flfl^EVH1^ and IVTT‐expressed ^35^S‐labelled wild‐type or mutated (FxxP to AxxA) (A) Prp16 and (B) Mira proteins. This experiment shows which of the two putative SLiMs of Prp16 or Mira are involved in the interaction. Figure is adapted from [8].

## Materials

### Production and immobilisation of the bait protein

1. Chemically competent SixPack [13] or other (e.g. BL21(DE‐3)) *E. coli* strain suitable for heterologous recombinant protein expression.
2. DNA plasmid encoding the GST‐fused POI (e.g. pGEX‐ or pDEST‐based plasmids).
3. Luria‐Bertani medium (LB broth): 10 g tryptone, 5 g NaCl and 5 g yeast extract in 1 L ddH_2_O, pH 7.2–7.5).
4. Isopropyl β‐D‐1‐thiogalactopyranoside (IPTG; Thermo Fisher Scientific cat#R0392, Waltham, MA, USA).
5. Carbenicillin‐Na_2_‐salt (Serva cat#15875.03, Heidelberg, Germany).
6. Phosphate‐buffered saline (PBS): 137 mm NaCl, 2.6 mm KCl, 8.7 mm Na_2_HPO_4_, 1.7 mm NaH_2_PO_4_.
7. Triton X‐100 (Sigma‐Aldrich cat#T9284, St. Louis, MO, USA).
8. PBST: PBS supplemented with 0.2% Triton X‐100.
9. Phenylmethylsulfonyl fluoride (PMSF; Sigma‐Aldrich cat#P7626).
10. Lysozyme (Sigma‐Aldrich cat#L‐6876).
11. Glutathione Sepharose 4B resin (Cytiva cat#17‐0756‐01, Washington, WA, USA).
12. Glycerol (Merck‐Millipore cat#1.04094.2500, Burlington, MA, USA).
13. Bovine serum albumin (BSA; Merck‐Millipore cat#1.12018.0100).
14. 125 and 300 mL Erlenmeyer flask and centrifuge tubes.
15. Laboratory rotator, refrigerated centrifuge.

### IVTT expression and labelling of the prey protein

1. Template DNA:
  1.1 Regular plasmid DNA: any type of plasmid vector that contains the cDNA or CDS‐encoding the protein/domain/fragment of interest under the regulation of the phage T7 promoter. This includes commercially available cDNA libraries, too.
  1.2 IVTT‐helper plasmid DNA: special types of plasmids that encode mRNA‐stabilising UTR sequences upstream and downstream of the protein/domain/fragment of interest under the regulation of the phage T7 promoter (e.g. pHY22 [14] or commercially available plasmids).
  1.3 PCR‐generated linear DNA: in this case, the linear DNA fragment encoding full‐length or truncated proteins is made by PCR using oligonucleotide primers to add 5′ T7 promoter, *Kozak consensus sequence*, initiation codon (ATG) and 3′ stop codons (Tips & Tricks 1 and 2).
2. Oligonucleotide primers:
  2.1 Forward:
    5'‐GAATTAATACGACTCACTATAGGGAGA *GCCGCCACC*ATG‐20‐mer gene‐specific sequence‐3'
  2.2 Reverse: 5'‐TCATTACTATCA‐20‐mer gene‐specific sequence‐3′ (Tips & Tricks 2)
3. High fidelity DNA polymerase kit (Phusion HF, NEB cat#M0530L, Ipswich, MA, USA).
4. Agarose (Lonza cat#50004, Basel, Switzerland).
5. Tris Acetate‐EDTA buffer (TAE): 400 mm Tris base, 1.14% acetic acid, 10 mm EDTA.
6. Gel extraction kit (Macherey‐Nagel cat#740609, Düren, Germany).
7. Plasmid DNA miniprep kit (Macherey‐Nagel cat#740588).
8. TnT T7 Quick Coupled Transcription/Translation System (Promega, cat#L1170, Madison, WI, USA) (Tips & Tricks 3).
9. Easy Tag Methionine‐L [35S] (Perkin Elmer cat# NEG709A001MC, Waltham, MA, USA) (Tips & Tricks 4).
10. 50 × EDTA‐free protease inhibitor cocktail (PIC; Roche, cat#11873580001, Basel, Switzerland).
11. RNasin Plus RNase Inhibitor (Promega cat#N2611).
12. Safe‐lock and low protein binding microcentrifuge tubes (Sarstedt cat#72.706.600, Nümbrecht, Germany).
13. Agarose gel‐electrophoresis system (Bio‐Rad cat#1704406, Berkeley, CA, USA).

### Pull‐down and autoradiography experiments

1. Buffers and solutions (Tips & Tricks 5):
  1.1 Binding buffer (BB): 50 mm HEPES pH 7.5, 150 mm NaCl, 2 mm MgCl_2_, 1 mm EGTA, 1 mm DTT, 0.1% Triton X‐100, 1xPIC and 0.5% BSA.
  1.2 Wash buffer 1 (WB1): BB without BSA
  1.3 Wash buffer 2 (WB2): WB1 supplemented with 50 mm NaCl and 0.1% Triton X‐100.
  1.4 Protein sample buffer: 62.5 mm TrispH 6.8, 10% glycerol, 2.3% SDS, 0.2% bromophenol blue, 5% 2‐mercaptoethanol.
  1.5 SDS/PAGE running buffer: 25 mm TrispH 8.3–8.5, 192 mm glycine, 0.1% SDS.
  1.6 Gel fixative solution: 10% acetic acid.
  1.7 Gel staining solution: 0.1% Coomassie Brilliant Blue‐R250 (VWR cat#M128, Radnor, PA, USA), 50% methanol, 10% acetic acid.
  1.8 Gel destaining solution: 10% methanol, 7% acetic acid.
  1.9 Transfer buffer (Bjerrum & Schafer‐Nielsen buffer with SDS): 48 mm Tris, 39 mm glycine, 20% methanol, 0.0375% SDS (pH 9.2) (Tips & Tricks 6).
  1.10 Ponceau S solution: 0.1% Ponceau S (Sigma‐Aldrich cat#P3500) in 5% acetic acid.
  1.11 X‐ray film developer (Tetenal cat#102408).
  1.12 X‐ray film fixative (Tetenal cat#102413).
2. Glycerol (Merck‐Millipore cat#1.04094).
3. Tris‐Glycine or Bis‐Tris SDS/PAGE gels.
4. Hypersensitive X‐ray film (CL‐XPosure Film, Thermo Fischer Scientific cat#34090 or BioMax MS Film, Carestream cat#1111681) (Tips & Tricks 7).
5. Cassettes for autoradiography.
6. Low energy intensifying screen for autoradiography (BioMax Transcreen LE) (Tips & Tricks 8).
7. Polyacrylamide gel‐electrophoresis (PAGE) system (Bio‐Rad cat# 1658000)
8. Semi‐dry protein transfer system (Bio‐Rad cat# 1703940) (Tips & Tricks 6).
9. Gel dryer (Bio‐Rad, Model 583) (Tips & Tricks 9).
10. Polyvinylidene fluoride (PVDF) membrane (Merck‐Millipore cat#IPVH00010) (Tips & Tricks 10).
11. Gel documentation system or flat scanner.

## Methods

### Bacterial expression of the bait protein (GST‐POI)

1. Transform chemically competent *E. coli* cells of choice with the GST alone (for negative control) and GST‐POI‐encoding plasmid DNA, respectively, using standard procedures. Plate the cells onto LB agar plates supplemented with selective antibiotics and grow at 37 °C for 16–18 h.
2. Starter culture: Inoculate 3–5 colonies into 25 mL LB broth (supplemented with 100 μg·mL^−1^ carbenicillin) in a 125 mL flask and shake at 37 °C for 16–18 h with 280 r.p.m. using orbital shaker with 19 mm diameter (or similar).
3. Add 500 μL starter culture into 50 mL LB broth (supplemented with 100 μg·mL^−1^ carbenicillin) in a 300 mL flask and grow at 37 °C to A600nm ~ 0.4–0.6.
4. Induce expression by adding 0.5–1 mm IPTG to the culture and shake cells at 37 °C for 3 h (Tips & Tricks 11).
5. Keep the flask on ice for 10 min and harvest cells by centrifugation at 3500 × ***g***, 4 °C for 15 min. Discard the media.
6. Resuspend cells in ice‐cold PBS and harvest by centrifugation at 3500 × ***g***, 4 °C for 15 min. Discard the supernatant. Flash‐freeze the cells in liquid nitrogen and store at −80 °C.

### Purification and immobilisation of GST and GST‐POI

1. Resuspend bacteria in 30–40 mL ice‐cold PBST supplemented with 200 μg·mL^−1^ lysozyme and 1 mm PMSF. Mix gently and keep on ice for 10 min.
2. Lyse the cells by sonication for 2 min (20 s pulse, 30 s break) on ice. Repeat this step 2–3 times until the suspension gets clear (Tips & Tricks 12).
3. Centrifuge the cell lysate at 15–21 000 × ***g*** at 4 °C for 15 min. Save the supernatant (clarified lysate).
4. Add 100–200 μL Glutathione Sepharose 4B beads into 10 mL PBST in a new 50 mL conical tube, mix gently and settle beads by centrifugation (600 × ***g*** at 4 °C for 3 min, with low deceleration to avoid turbulence). Carefully discard or aspirate off the buffer.
5. Pour the clarified lysate from step 3 onto the beads and mix the sample for 1 h at 4 °C with gentle rotation (binding).
6. Settle the beads by centrifugation as in step 4 and discard the supernatant.
7. Add 40 mL PBST to the beads and mix for 5 min at 4 °C with gentle rotation (washing). Settle the beads by centrifugation and discard the supernatant. Repeat this step 3 times.
8. Resuspend the beads in 1 mL PBS supplemented with 50% glycerol. Store the beads at −20 °C until use (Tips & Tricks 13).

### Determining the amount of bait immobilised to beads

1. Gently mix the tube with immobilised GST or GST‐POI and take out 50 μL suspension.
2. Add to 1 mL PBST and settle beads by centrifugation (see Purification and immobilisation of GST and GST‐POI/step 4).
3. Aspirate off the supernatant (Tips & Tricks 14).
4. Mix the beads with 50 μL protein sample buffer and boil for 3 min (elution). Centrifuge at 12,000 × ***g*** for 5 min at room temperature (RT).
5. Run 1, 2, 4, 8 and 16 μL of supernatants with 1, 2, 4, 8, 16 μg BSA standards on SDS/PAGE.
6. Incubate the gel in fixative solution for 10 min at RT with gentle shaking.
7. Incubate the gel in staining solution for 12 min at RT with gentle shaking.
8. Incubate the gel in destaining solution for 1–4 h at RT with gentle shaking.
9. Estimate the amount of GST and GST‐POI by comparing the band intensities with the BSA standards.

### IVTT expression and labelling of prey proteins

1. Set up the following reaction in safe‐lock microcentrifuge tubes on ice:
  16 μL TnT T7 Quick Coupled Transcription/Translation reagent;
  0.4 μL 50 × PIC;
  0.4 μL RNasin Plus RNase Inhibitor;
  0.8 μL Methionine‐L [35S] (Tips & Tricks 4).
2. Add template DNA and mix gently:
  50–100 ng plasmid DNA or
  30–60 ng purified PCR product and
  0.4 μL PCR enhancer (included in the kit).
3. Add nuclease‐free water to 25 μL. Mix gently.
4. Incubate the reaction mixture at 30 °C for 1 h.
5. Centrifuge at 12 000 × ***g*** for 5 min at RT.
6. Save the supernatant and use immediately or store at −20 °C (max 1 month).
7. Take 1 μL, mix with 9 μL sample buffer and boil for 3 min (IVTT input).

### Pull‐down experiment

1. Take 1–5 μg GST or GST‐POI containing beads (see Methods/Purification and immobilisation of GST and GST‐POI/step 8 and Methods/Determining the amount of bait immobilised to beads/step 9), respectively, and wash in 1 mL BB buffer (see Materials/Pull‐down and autoradiography experiments/step 1). Settle beads by centrifugation (600 × ***g*** at 4 °C for 3 min, low deceleration).
2. Completely remove the supernatant (Tips & Tricks 14) and resuspend the beads in 800 μL BB buffer.
3. Add 10 μL ^35^S‐labelled prey (see Methods/IVTT expression and labelling of prey proteins/step 6) into the GST and GST‐POI suspension, respectively.
4. Incubate the binding reaction for 60–90 min at 4 °C. Mix gently on a rotating wheel (Tips & Tricks 15).
5. Settle beads (containing the putative complex of the immobilised bait and ^35^S‐labelled prey proteins) by centrifugation and discard the supernatant (unbound proteins) into a waste container suitable for isotope storage.
6. Wash the beads with 1 mL WB1 (see Materials/Pull‐down and autoradiography experiments/step 1) for 3 min at 4 °C with gentle rotation. Settle the beads by centrifugation and discard the supernatant. Repeat this step twice.
7. Add 1 mL WB2 (see Materials/Pull‐down and autoradiography experiments/step 1) to the beads, resuspend by carefully pipetting up/down and mix for 3 min at 4 °C with gentle rotation. Settle the beads by centrifugation and discard the supernatant. Repeat this step twice (Tips & Tricks 14).
8. Resuspend beads in 12 μL protein sample buffer, boil for 5 min (elution) and centrifuge at 12 000 × ***g*** for 5 min at RT.

### SDS/PAGE and autoradiography

1. Load a molecular weight marker, the IVTT input (5–10 μL), GST (10 μL) for negative control and GST‐POI pull‐downs (10 μL) into SDS/PAGE.
2. Disassemble the gel and either:
  2.1 stain the gel with Coomassie Brilliant Blue and differentiate as described in Methods (see Determining the amount of bait immobilised to beads/steps 6–8), rinse 3 times in ddH_2_O, digitalize and dry using a gel dryer;
  2.2 or blot the proteins to PVDF membrane using a semidry transfer system (Tips & Tricks 6), rinse the membrane in ddH_2_O, stain with Ponceau S solution for 10 min, rinse in ddH_2_O, digitalize and air dry for 10 min.
3. Put the dried gel/PVDF membrane into the cassette and secure it with adhesive tape. Place a hypersensitive film onto the gel/membrane in dark (Tips & Tricks 7 and 8). Lock the cassette (Tips & Tricks 16).
4. Perform exposure at −80 °C for 6–48 h. Shorter and longer exposures may apply.
5. In a dark room carefully remove the film from the cassette, wave it few times to warm it up and:
  5.1 put it into an automated film developing machine;
  5.2 or put it into room temperature X‐ray film developing solution (see Materials/Pull‐down and autoradiography experiments/steps 1.11–12.) for 60 s. Shake the film gently. Rinse in water and fix for 1–2 min.
6. Air dry the film and label protein ladders. Digitalize the film.

## Tips & Tricks

1. Add 4 consecutive stop codons to the 3′‐end of the PCR product. If the truncated prey does not contain methionine, add an extra Met‐Met‐Gly sequence to the C‐terminus of the protein (add an extra ATGATGGGGT‐4xStop to the 3′ end of the DNA).
2. PCR‐generated linear DNA fragments are suitable for the IVTT expression of polypeptides ranging from 9.5 to 250 kDa.
3. Other types of IVTT reagents are also available from various vendors. In some cases, SP6 promoter is better than T7 and wheat germ or bacterial IVTT systems provide a higher yield.
4. Sulphur‐35 is a low‐energy beta‐emitter. Although its radiation barely penetrates the gloves and the skin of the person, extra care needs to be taken when working with ^35^S. The experimenter must follow institutional regulations and must have permission to work with radionuclides. In some cases, a separate hot lab or bench as well as protecting and decontaminating equipment/reagents are required to work with ^35^S. An alternative to radioactive labelling, biotin‐ or fluorescently‐tagged lysine can also be used in IVTT.
5. Other types of buffers (salts, pH, cofactors, reducing agents, etc.) can also be used, which need to be optimised empirically or taken from previously published articles. For more details see [15, 16] or Buffers. Do not use EDTA! Nonionic surfactants (Triton X‐100, Tween‐20 or NP‐40) are recommended to avoid nonspecific binding to the beads or plastic ware.
6. Semi‐dry protein transfer (e.g. in Bjerrum & Schafer‐Nielsen buffer) is more efficient than conventional wet transfer for autoradiography. If other means of transfer is employed, use the appropriate buffer.
7. These films are extremely light sensitive; therefore, film development must be carried out in a dark room without any helper light.
8. A low energy intensifying screen between the gel and the film lowers the exposure time and increases the resolution of the autoradiography image. If the screen is not available, the exposure time should be extended (e.g. doubled).
9. If the gel dryer is not available, blot the proteins to PVDF, stain with Ponceau S, take a photo, air dry the membrane for 10 min and expose it to hypersensitive film.
10. We recommend the usage of PVDF instead of nitrocellulose because nitrocellulose gets fragile at −80 °C.
11. If the bait protein forms an insoluble inclusion body, lower the temperature to 18–24 °C, use less IPTG (0–0.1 mm) for induction and extend the expression time for up to 24 h. Alternatively, auto‐induction media can also be used. If the protein level is low, use Terrific Broth instead of LB broth.
12. Other types of cell disruption methods can also be applied, including chemical lysis, emulsion flex, freezing techniques, etc. If the GST‐POI is insoluble, use the Sarkosyl method to purify GST and GST‐POI according to [17].
13. Storing the immobilised bait proteins in PBS with 50% glycerol at −20 °C prevents the GST and GST‐POI from freezing, contamination and degradation. Beads can be stored under these conditions for years.
14. To avoid losing the beads, use G25 or G26 needle attached to the aspirator or a syringe.
15. Binding reaction is normally performed at lower temperatures. However, in some cases, it can be done at a higher temperature, which needs to be optimised for each experiment.
16. If available, a phosphorimager device (e.g. Cytiva Typhoon) with low energy screen cassette can also be used (in this case Methods/SDS/PAGE and autoradiography/steps 3–6 are not needed). Although this equipment shortens (minutes to hours) and simplifies the procedure, the resolution of the image will be lower. On the other hand, it generates digital images with a much wider dynamic range compared with X‐ray films.

## Conflict of interest

The authors declare no conflict of interest.

## Author contributions

ZR‐N, EA and ZL developed the protocol and wrote the paper. ZL made and edited the figures.

## Data Availability

The data presented in the figures (Figs 2, 3 and 4) of this paper were previously published in Open Access journals [5, 8, 11] distributed under the terms of the Creative Commons CC BY licence. This permits unrestricted use, distribution and reproduction of the figures in any medium, provided the original work is properly cited.
